# MHF-Net: a multi-modal hybrid fusion network for ophthalmic disease diagnosis

**DOI:** 10.3389/fmed.2026.1879177

**Published:** 2026-08-17

**Authors:** Zhiming Xiong, Meiting Yu, Tong Lv, Ruixuan Xu, Chongjun Huang, Guohui Yuan, Zhuoran Wang

**Affiliations:** 1 School of Information and Communication Engineering, University of Electronic Science and Technology of Chengdu, Chengdu, Sichuan, China; 2 Department of Nephrology, The Quzhou Affiliated Hospital of Wenzhou Medical University, Quzhou People's Hospital, Quzhou, Zhejiang, China; 3 Clinical Medical College, Hainan Medical University, Haikou, Hainan, China; 4 Yangtze Delta Region Institute (Quzhou), University of Electronic Science and Technology of China, Quzhou, Zhejiang, China

**Keywords:** color fundus photography, feature enhancement, fundus disease diagnosis, multimodal fusion, Optical Coherence Tomography

## Abstract

Fundus diseases are a leading cause of irreversible blindness worldwide. Existing Color Fundus Photography (CFP) and Optical Coherence Tomography (OCT) fusion methods face two major challenges: feature noise interference and the semantic gap between heterogeneous modalities, leading to suboptimal fusion and limited diagnostic accuracy. To address these issues, this paper proposes a Multi-modal Hybrid Fusion Network (MHF-Net), which is designed to suppress noise, enhance intra-modal feature representation, and explicitly establish cross-modal semantic correlations. The proposed framework consists of three core components: a heterogeneous dual-stream encoder to extract multi-level pathological features from both CFP and OCT; a Multi-scale Feature Enhancement (MFE) module to reduce background noise while reinforcing intra-modal features; and a Hierarchical Contextual Fusion (HCF) module to explicitly establish deep semantic correlations across modalities and scales. Evaluated on the Topcon-MM dataset, MHF-Net achieves a Macro F1 of 80.91%, a Macro Recall of 76.80%, and a Sample Accuracy of 67.32%, demonstrating competitive performance against state-of-the-art multimodal models and recent vision foundation model baselines. This study demonstrates that explicit hierarchical fusion with multi-scale feature enhancement effectively resolves the semantic gap between CFP and OCT modalities, providing a robust and clinically promising solution for automated multimodal diagnosis of complex fundus diseases.

## 1 Introduction

Retinal diseases, such as cataracts, diabetic retinopathy (DR), and age-related macular degeneration (AMD), have become the leading causes of visual impairment and irreversible blindness worldwide (1–3). Early screening and precise diagnosis are essential for effective treatment plans.

In recent years, deep learning has achieved significant progress in ophthalmic image analysis (4). Early research primarily focused on single-modality diagnosis, utilizing Convolutional Neural Networks (CNNs) to extract features from either CFP or OCT individually (5, 6). While these approaches have demonstrated promising performance, they are inherently limited by relying on a single source of information, making it difficult to capture complex pathological characteristics of challenging cases.

In clinical practice, different imaging modalities provide complementary diagnostic information. Color Fundus Photography (CFP) provides high-resolution two-dimensional (2D) en-face projections, clearly revealing hemorrhages, exudates, and vascular morphology (7); however, it lacks depth information necessary to assess deep retinal lesions. Conversely, Optical Coherence Tomography (OCT) captures micron-scale cross-sectional views that are highly sensitive to intra-layer fluid and thickness variations (8), yet it lacks an overall fundus perspective.

To exploit this complementarity, multimodal learning has gradually become the dominant paradigm in ophthalmic diagnosis. Early approaches mainly relied on decision-level fusion or naive feature concatenation. For example, Yoo et al. (9) independently extracted features using VGG-19 and classified them using Random Forests, demonstrating the benefit of multimodal information in AMD diagnosis. Wang et al. (10) further proposed a dual-stream CNN with loose pairing strategy to address limited availability of strictly aligned clinical data. Subsequent methods attempted to improve feature representation capability; for instance, M2LC-Net (11) incorporated CBAM (12) to enhance discriminative features before fusion.

More recently, hierarchical and multi-level fusion strategies have been explored to better model cross-modal relationships. Li et al. (13) compared early, intermediate, and hierarchical fusion strategies and demonstrated the superiority of hierarchical fusion in handling modality complementarity. Nevertheless, most existing methods still rely on simple operations such as concatenation or addition, which are insufficient to model deep nonlinear semantic interactions. In addition, significant spatial misalignment and semantic gaps between CFP and OCT remain insufficiently addressed, often leading to suboptimal cross-modal alignment. Another critical issue is modality imbalance, where models tend to over-rely on the more dominant modality while suppressing the contribution of the other.

Beyond fusion strategies, multi-scale representation learning has also been widely studied due to the large variation in lesion sizes, ranging from microaneurysms to large retinal detachments. He et al. proposed MSAN (14), which integrated multi-scale attention mechanisms to capture pathological regions at different resolutions. Song et al. (15) introduced an information bottleneck strategy to reduce redundancy in multi-scale features. Chen et al. (16) further proposed vertical plane feature fusion (VPFF) to combine structural OCT information with global retinal context. However, most existing multi-scale methods directly aggregate features without explicitly filtering noise from low-level representations, which may introduce non-pathological artifacts and degrade diagnostic performance.

Attention mechanisms have also been widely adopted to enhance feature representation. SE-Net (17) performed channel-wise recalibration, while CBAM (12) further incorporated spatial attention for fine-grained enhancement. In multimodal settings, attention mechanisms are increasingly used to model inter-modal interactions. For example, TFA-Net (18) introduced reverse cross-attention to enhance CFP and SS-OCTA representations, while M3 (19) combined self-attention and cross-modal attention to capture latent modality relationships. CRD-Net (20) further strengthened cross-modal dependencies through dedicated attention modules. With the development of Transformer architectures, more advanced multimodal interaction models have emerged, such as MMRAF (21), which integrated hierarchical attention and contrastive alignment for robust representation learning.

Despite these advances, existing attention-based fusion strategies are still largely constrained to single-scale or shallow interaction modeling, making it difficult to fully capture cross-scale and cross-modal semantic dependencies.

Based on the above literature review, several critical research gaps remain in existing CFP–OCT multimodal fusion methods.

- **Gap 1: Lack of effective noise suppression before multi-scale fusion**. Existing multi-scale fusion methods directly aggregate low-level and high-level features without explicitly suppressing background noise, which may introduce non-pathological artifacts and degrade feature discriminability.
- **Gap 2: Oversimplified cross-modal fusion strategies**. Most existing methods rely on simple operations such as concatenation or element-wise addition, which are insufficient to model the complex non-linear interactions between heterogeneous modalities such as CFP and OCT.
- **Gap 3: Limited depth of cross-modal interaction**. Current attention-based fusion approaches are often restricted to single-scale or shallow interactions, failing to fully exploit cross-scale semantic dependencies between global structural information and local lesion details.
- **Gap 4: Limited exploration of CFP–OCT multimodal learning for comprehensive ophthalmic diagnosis**. Although recent studies have achieved promising results in single-modal ophthalmic tasks (22–24), such as retinal vessel segmentation and diabetic retinopathy classification, these methods remained limited in multimodal settings and did not address key challenges including cross-modal semantic alignment, modality imbalance, and multi-label disease co-occurrence modeling.

These limitations suggest that an effective CFP–OCT fusion framework should jointly address modality-specific feature robustness, explicit noise suppression, and progressive cross-modal interaction at multiple semantic levels. In contrast to representative multimodal ophthalmic models such as CRD-Net, MMRAF, and M3, which mainly relied on homogeneous encoders and shallow or single-scale fusion strategies, the proposed MHF-Net adopts a heterogeneous dual-stream design together with multi-scale feature enhancement and hierarchical contextual fusion. This design enables more effective modality-aware representation learning and cross-scale semantic alignment, thereby providing a more robust framework for multi-label fundus disease diagnosis.

Motivated by the above limitations, this paper proposes a Multi-modal Hybrid Fusion Network (MHF-Net) for CFP and OCT-based ophthalmic disease diagnosis. The main contributions are summarized as follows:

- **MHF-Net framework:** We propose an end-to-end dual-stream architecture for joint CFP and OCT analysis, providing a unified framework for multimodal fundus disease diagnosis.
- **Modality-aware feature learning and enhancement:** We design a heterogeneous encoding and feature enhancement strategy to improve the robustness of single-modality representations, effectively suppressing noise and enhancing the discriminability of subtle lesion features.
- **Hierarchical cross-modal fusion mechanism:** We introduce a progressive fusion strategy that models cross-modal interactions across multiple levels, enabling effective semantic alignment between global CFP structures and local OCT details.

The remainder of this paper is organized as follows. Section 2 describes the proposed MHF-Net framework, including the dual-stream heterogeneous encoder, the multi-scale feature enhancement (MFE) module, and the hierarchical contextual fusion (HCF) module. Section 3 presents the experimental results, including dataset description, implementation details, evaluation metrics, comparative experiments, and ablation studies. Section 4 discusses the findings and provides visualization analysis. Section 5 concludes the paper and outlines future work.

## 2 Methods

### 2.1 Overall framework

**Figure 1:**
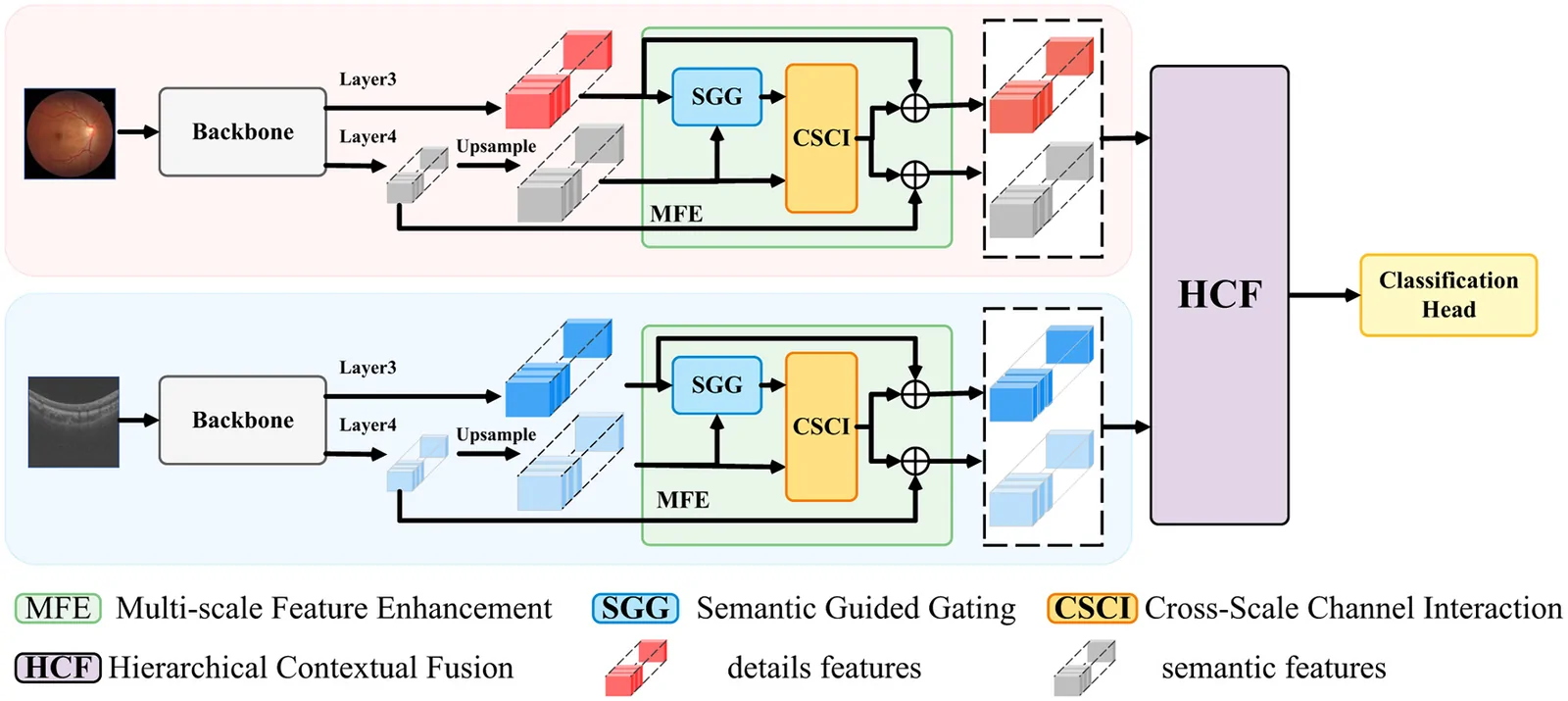
Overall architecture of MHF-Net.

In this study, we propose a dual-stream diagnostic framework named the Multi-modal Hybrid Fusion Network (MHF-Net). The network is designed to leverage the complementary information between CFP and OCT for precise classification of fundus diseases. The overall architecture of MHF-Net is illustrated in Figure 1.

Specifically, the Dual-Stream Heterogeneous Encoder (DHE) employs two independent backbone networks to extract multi-level feature representations from CFP and OCT images, respectively. The resulting modality-specific features are then refined by the Multi-scale Feature Enhancement (MFE) module, which suppresses irrelevant noise and enhances lesion-related responses to obtain more discriminative representations. Subsequently, the Hierarchical Contextual Fusion (HCF) module performs progressive cross-modal interaction and multi-scale contextual aggregation, enabling effective integration of complementary information from the two modalities. Finally, the fused representation is fed into a classification head to generate multi-label diagnostic predictions.

The design of MHF-Net is motivated by two key challenges in multimodal fundus disease diagnosis: (1) modality-specific noise and weak lesion responses within individual modalities; (2) semantic discrepancies between CFP and OCT representations.

To address these challenges, MHF-Net adopts a progressive learning strategy. The DHE module first preserves modality-specific characteristics through independent feature extraction. The MFE module then enhances lesion-related representations while suppressing irrelevant background information. Finally, the HCF module progressively aligns and integrates complementary information across modalities and feature hierarchies, producing a unified representation for diagnosis.

### 2.2 Dual-stream feature encoding

Given the heterogeneous nature of CFP and OCT images, a dual-stream heterogeneous encoder architecture is adopted for feature extraction. CFP and OCT are generated from different imaging principles and provide complementary diagnostic information. CFP primarily reflects retinal appearance, vascular structures, and lesion distributions, whereas OCT captures cross-sectional retinal morphology and layered tissue structures. As a result, the feature distributions and visual patterns of the two modalities differ substantially. To better accommodate these modality-specific characteristics and maximize complementary information extraction, independent backbone networks are employed for CFP and OCT feature encoding. Specifically, the CFP branch adopts ResNet-50 to strengthen the modeling of global morphological structures and lesion areas. Conversely, the OCT branch utilizes ConvNeXt (25) to enhance the representation of retinal layered structures and fine-grained local variations.

Considering the significant spatial scale variations of fundus lesions, single-scale deep features often struggle to balance global semantics with local details. Therefore, we extract intermediate-layer (Layer 3) and deep-layer (Layer 4) features from the backbone networks, corresponding to low-level detail information and high-level semantic representations, respectively. This hierarchical multi-scale feature representation effectively integrates shallow spatial structural information with deep abstract semantics.

For a given input of a CFP image *X*_*cfp*_ and an OCT image *X*_*oct*_, the backbone network E is used to extract features as defined in Equations 1, 2:


$$\begin{array}{l} F_{cfp}^{3},F_{cfp}^{4}=E_{cfp}\left(X_{cfp}\right) \end{array}$$ (1)

$$\begin{array}{l} F_{oct}^{3},F_{oct}^{4}=E_{oct}\left(X_{oct}\right) \end{array}$$ (2)

where $F_{m}^{l}$ denotes the feature representation of modality *m* ∈ {*cfp, oct*} at the *l*-th *l* ∈ {3, 4} layer. Specifically, $F_{m}^{3}\in R^{C_{3}\times H_{3}\times W_{3}}$ contains rich textural details, while $F_{m}^{4}\in R^{C_{4}\times H_{4}\times W_{4}}$ encapsulates high-level semantic information.

### 2.3 Multi-scale feature enhancement module

**Figure 2:**
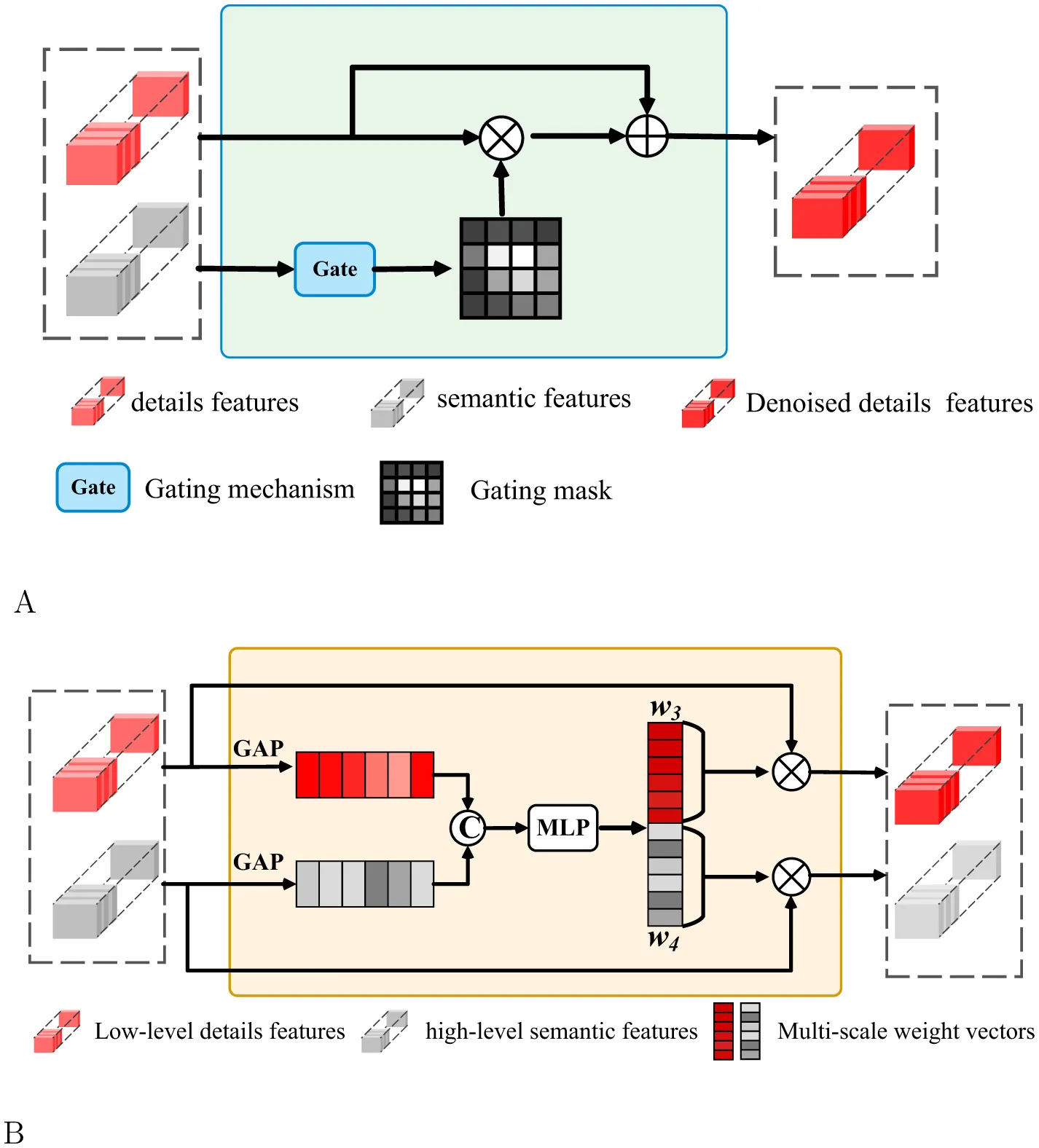
**(A)** Semantic-Guided Gating (SGG) module. **(B)** Cross-Scale Channel Interaction (CSCI) module.

Directly fusing raw backbone features may introduce noisy or weakly discriminative information into the multi-modal fusion process, especially for subtle retinal lesions. Therefore, feature enhancement is performed prior to cross-modal interaction to improve the quality of modality-specific representations. We introduce a Multi-scale Feature Enhancement (MFE) module, which aims to suppress background noise and enhance discriminative lesion features across different scales. As illustrated in Figure 2, MFE consists of two components: Semantic-Guided Gating (SGG) and Cross-Scale Channel Interaction (CSCI).

#### 2.3.1 Semantic-guided gating

The intuition behind SGG is that high-level semantic features provide reliable lesion localization cues, which can be used to guide the enhancement of low-level detailed representations. Specifically, $F_{m}^{4}$ and $F_{m}^{3}$ *m* ∈ {*cfp, oct*} are first aligned to a unified channel dimension (C = 512) via 1 × 1 convolutions. Then, $F_{m}^{4}$ is upsampled to the same spatial resolution as $F_{m}^{3}$ using bilinear interpolation. Subsequently, a lightweight gating convolutional network generates the gating mask M, as defined in Equation 3:


$$\begin{array}{l} {\text{M}}_{m}=\sigma \left(G\left(Up\left(F_{m}^{4}\right)\right)\right) \end{array}$$ (3)

Where σ() denotes the Sigmoid activation function, *G* represents the gating mapping module consisting of a 1 × 1 convolutional layer, Batch Normalization (BN), and ReLU activation, and *Up*() denotes bilinear interpolation upsampling. Finally, the detail features are enhanced through a residual connection, as defined in Equation 4:


$$\begin{array}{l} {\hat{F}}_{m}^{3}=F_{m}^{3}⊙\left(1+{\text{M}}_{m}\right) \end{array}$$ (4)

It is worth noting that the residual attention adopted in this study not only ensures the stable propagation of gradient flow from the original detail features—preventing information loss caused by over-suppression from the gate—but also specifically reinforces the salient pathological regions indicated by the deep semantics. Consequently, SGG effectively mitigates the interference of background noise on subtle lesion features in multi-label classification tasks.

#### 2.3.2 Cross-scale channel interaction mechanism

Although SGG enhances spatially salient regions, the channel responses of shallow and deep features may still focus on different semantic concepts. To address this, the CSCI module is introduced to promote semantic consistency across feature hierarchies. Specifically, CSCI first applies Global Average Pooling (GAP) to the SGG-enhanced feature ${\hat{F}}_{m}^{3}$ and the deep feature $F_{m}^{4}$. The resulting channel descriptors are then concatenated as defined in Equation 5:


$$\begin{array}{l} v_{m}=\left[\text{GAP}\left({\hat{F}}_{m}^{3}\right),\text{GAP}\left(F_{m}^{4}\right)\right] \end{array}$$ (5)

Subsequently, the concatenated global channel representation is fed into a Multi-layer Perceptron (MLP) to learn the joint cross-scale channel weights $w\in R^{C_{3}+C_{4}}$. These weights are then split along the channel dimension into weight vectors *w*_3_ and *w*_4_, corresponding to the two scales, as defined in Equation 6:


$$\begin{array}{l} \left[w_{m}^{3},w_{m}^{4}\right]=Split\left(\text{MLP}\left(v_{m}\right)\right) \end{array}$$ (6)

Finally, the features are recalibrated using these weight vectors to obtain the enhanced representations, as defined in Equations 7, 8:


$$\begin{array}{l} F_{m\text{\_}en}^{3}={\hat{F}}_{m}^{3}⊗w_{m}^{3} \end{array}$$ (7)

$$\begin{array}{l} F_{m\text{\_}en}^{4}=F_{m}^{4}⊗w_{m}^{4} \end{array}$$ (8)

### 2.4 Hierarchical contextual fusion module

Traditional fusion methods often relied on simple single-level concatenation, which failed to capture complex non-linear interactions across modalities and feature hierarchies, thereby limiting their ability to bridge the semantic gap between heterogeneous modalities. To address this issue, we propose a Hierarchical Contextual Fusion (HCF) module that enables progressive cross-modal interaction and multi-level contextual integration, facilitating more effective complementary fusion across modalities.

**Figure 3:**
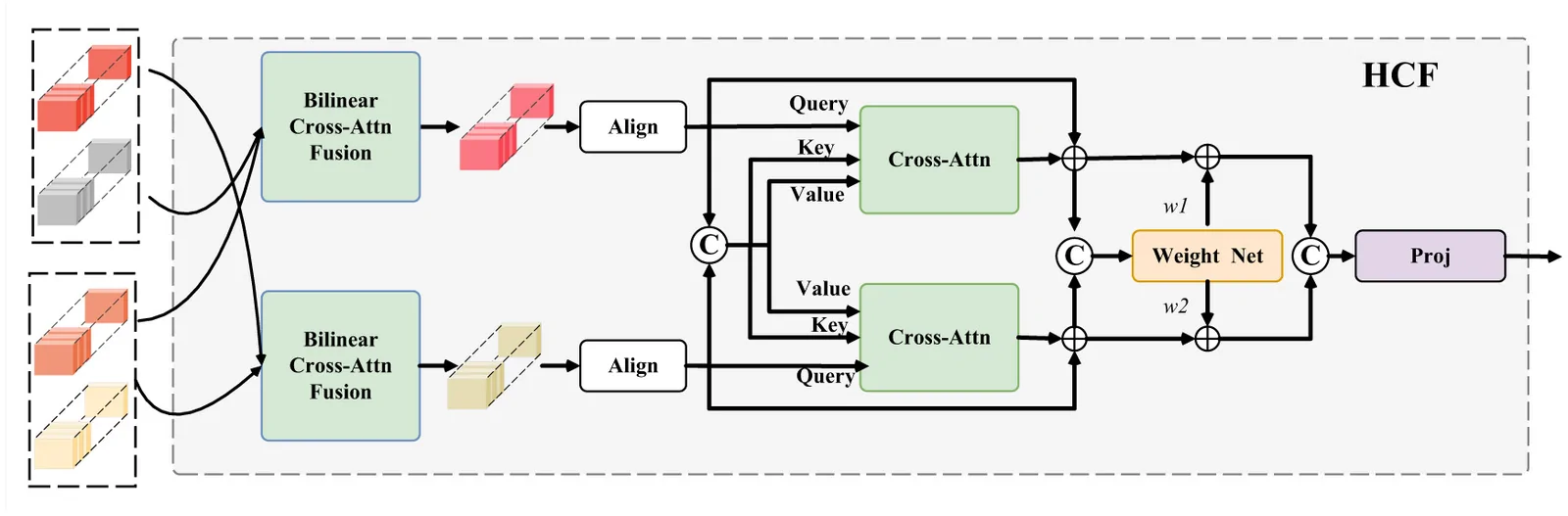
Detailed structure of the HCF module.

As illustrated in Figure 3, the HCF module consists of two components: intra-layer cross-modal interaction and inter-layer global interaction.

#### 2.4.1 Intra-layer cross-modal interaction

For each hierarchy *l* ∈ {3, 4}, we employ bidirectional cross-attention to fuse CFP and OCT features at the same level. Specifically, CFP features query the OCT context, and vice versa, thereby capturing local complementarity between modalities. To ensure gradient stability during training, residual connections and Layer Normalization (LN) are incorporated.

CFP Branch Enhancement (where CFP serves as the Query and OCT as the Key-Value pair) as defined in Equations 9, 10:


$$\begin{array}{l} H_{cfp}=\text{LN}\left(\text{MHA}\left(Q={\tilde{F}}_{cfp},K={\tilde{F}}_{oct},V={\tilde{F}}_{oct}\right)+{\tilde{F}}_{cfp}\right) \end{array}$$ (9)

$$\begin{array}{l} \text{MHA}\left(Q,K,V\right)=\text{Softmax}\left(\frac{QK^{T}}{\sqrt{d_{k}}}\right)V \end{array}$$ (10)

OCT Branch Enhancement (where OCT serves as the Query and CFP as the Key-Value pair) as defined in Equation 11:


$$\begin{array}{l} H_{oct}=\text{LN}\left(\text{MHA}\left(Q={\tilde{F}}_{oct},K={\tilde{F}}_{cfp},V={\tilde{F}}_{cfp}\right)+{\tilde{F}}_{oct}\right) \end{array}$$ (11)

The post-interaction features are processed by independent output projection layers for linear transformation. Subsequently, they are concatenated along the channel dimension and mapped to the final feature space through a fusion layer to obtain the fused representation of the current hierarchy, as defined in Equations 12, 13:


$$\begin{array}{l} P_{cfp}=W_{out}^{cfp}H_{cfp},\;P_{oct}=W_{out}^{oct}H_{oct} \end{array}$$ (12)

$$\begin{array}{l} Z_{fused}=\text{LN}\left(W_{fuse}\left[P_{cfp},P_{oct}\right]\right) \end{array}$$ (13)

Where *W*_*out*_ and *W*_*fuse*_ are learnable linear mapping weight matrices, and *Z*_*fused*_ represents the fused feature vector output.

#### 2.4.2 Inter-layer global interaction

While intra-layer interaction aligns modalities at the same scale, features across different hierarchies remain uncorrelated, limiting global contextual modeling. To address this, we introduce an inter-layer global interaction mechanism to capture dependencies across feature levels.

Specifically, the fused features *Z*_*l*_ from each layer are first projected into a unified dimension ${\tilde{Z}}^{l}=\phi_{align}^{l}\left(Z_{fused}^{l}\right)$. The feature sequence from all levels is then treated as a collective whole $S=\left[{\tilde{Z}}^{3},{\tilde{Z}}^{4}\right]$, which is modeled via a self-attention mechanism. This process captures global dependencies between different hierarchies, enabling the model to simultaneously synthesize local detail information and high-level semantic structures, as defined in Equation 14:


$$\begin{array}{l} {Ẑ}^{l}=\text{MHA}\left(Q={\tilde{Z}}^{l},K=S,V=S\right)+{\tilde{Z}}^{l} \end{array}$$ (14)

where MHA(·) denotes the multi-head attention operator, and *Q*, *K*, and *V* represent the query, key, and value matrices, respectively. $S=\left[{\tilde{Z}}^{3},{\tilde{Z}}^{4}\right]$ denotes the concatenated multi-level feature sequence, where ${\tilde{Z}}^{l}$ is the aligned feature representation at the *l*-th hierarchy and Ẑ^*l*^ denotes the globally enhanced representation for the *l*-th layer after inter-layer interaction.

Building upon this, we introduce a learnable weighted fusion network *g*(·), comprising a MLP and a Softmax function. This network adaptively assigns weights α_*l*_ to different levels based on the input features and performs weighted aggregation to produce the final multi-modal global representation *F*_*fused*_, as defined in Equation 15:


$$\begin{array}{l} \left[\alpha_{3},\alpha_{4}\right]=\text{Softmax}\left(\text{MLP}\left(\left[{Ẑ}^{3},{Ẑ}^{4}\right]\right)\right) \end{array}$$ (15)

where α_3_ and α_4_ are normalized scalar weights indicating the contribution of each hierarchy as defined in Equation 16.


$$\begin{array}{l} F_{fused}=\psi_{proj}\left(\left[\alpha_{3}\cdot {Ẑ}^{3},\alpha_{4}\cdot {Ẑ}^{4}\right]\right) \end{array}$$ (16)

where ψ_*proj*_(·) denotes a learnable projection layer that maps the weighted concatenated features into the final multimodal representation space, and *F*_*fused*_ represents the final fused feature output.

## 3 Result

### 3.1 Dataset description and pre-processing analysis

In this study, the TOPCON-MM dataset (26) is utilized to validate the effectiveness of the proposed model. TOPCON-MM is a multi-modal ophthalmic imaging dataset comprising CFP and OCT images from 369 eyes of 203 patients (Table 1). The original resolution of the CFP images is 2,576 × 1,934 pixels, while the OCT scans range from 320 × 992 to 1,024 × 992 pixels. Each eye contains multiple CFP images and corresponding OCT B-scans, and may be associated with multiple pathological labels, making it a typical multi-label classification task.

**Table 1:** Statistics of the Topcon-MM dataset.

Category	Normal	dAMD	wAMD	DR	CSC	PED	MEM	FLD	EXU	CNV	RVO	Total
Eyes	153	52	30	72	15	23	38	93	90	14	10	369
CFP	299	178	171	502	95	134	200	638	576	143	34	1,520
OCT	278	160	145	502	95	133	196	613	573	137	34	1,435

The dataset contains a total of 11 categories, including normal, dry age-related macular degeneration (dAMD), wet age-related macular degeneration (wAMD), diabetic retinopathy (DR), central serous chorioretinopathy (CSC), pigment epithelial detachment (PED), macular epiretinal membrane (MEM), fluid (FLD), exudation (EXU), choroid neovascularization (CNV), and retinal vascular occlusion (RVO).

To avoid data leakage caused by the “one-to-many” mapping (one CFP image corresponding to multiple OCT B-scans), a patient-level split strategy is adopted: images are grouped by Patient ID, and a greedy stratified sampling algorithm is used to assign patient groups to training and validation sets (target ratio 8:2) while maintaining patient isolation.

**Figure 4:**
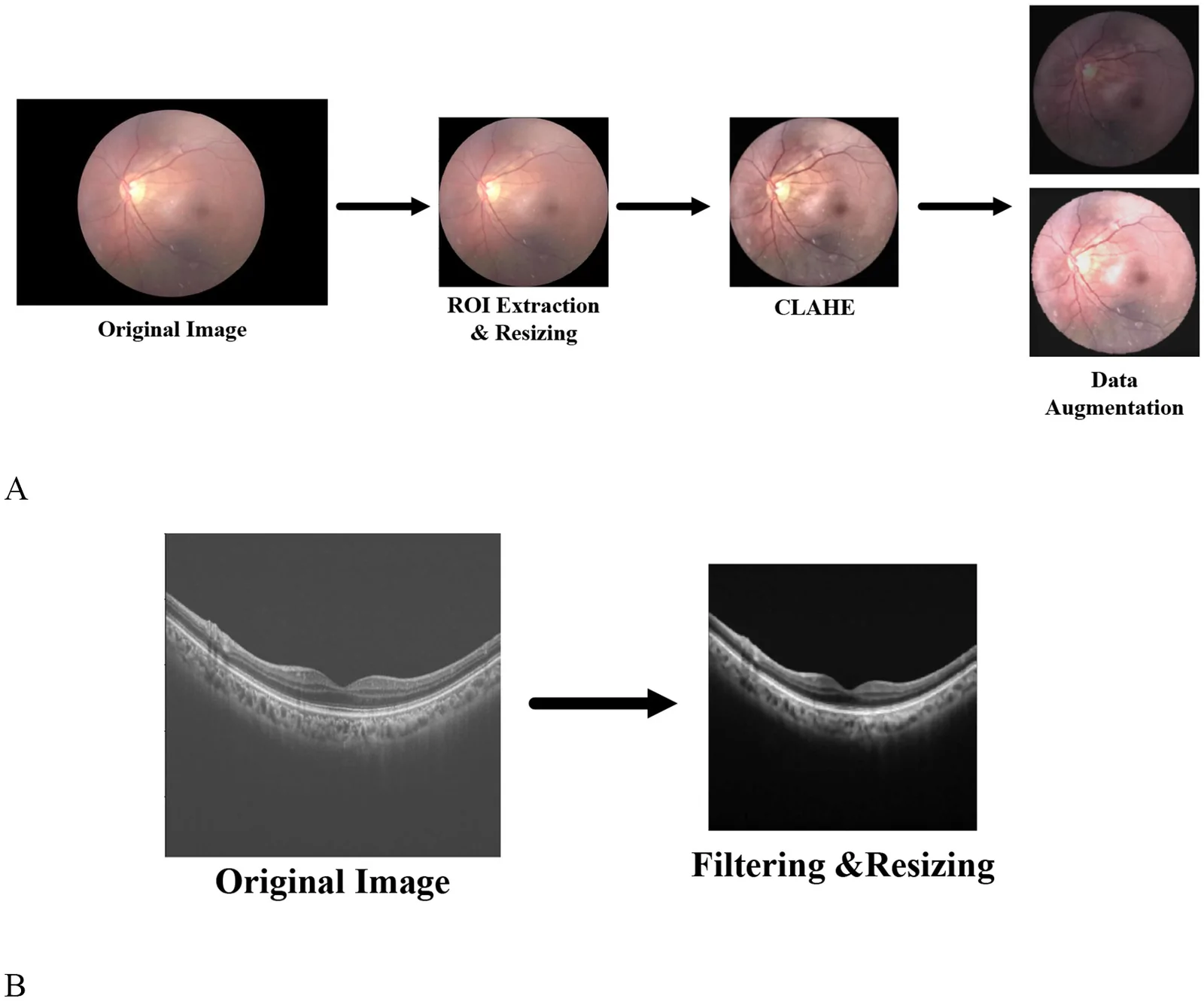
Image preprocessing results. **(A)** CFP images. **(B)** OCT images.

To standardize the input distribution and enhance feature representation, a unified preprocessing pipeline is adopted, as illustrated in Figure 4. For CFP images, region-of-interest extraction and Contrast Limited Adaptive Histogram Equalization (CLAHE) are applied to reduce background interference and improve lesion visibility, while OCT images are denoised to suppress speckle noise. All images are then normalized and resized to 224 × 224 pixels to meet the input requirements of the backbone networks.

During training, data augmentation strategies, including random cropping, flipping, rotation, and color jittering (for CFP only), are employed to improve model generalization and robustness. No augmentation is applied during validation and testing.

### 3.2 Implementation details

To ensure the reproducibility and fairness of the experimental results, all comparative experiments utilize a consistent hyperparameter setting. The specific training strategies are detailed in Table 2.

**Table 2:** Hyperparameters and training strategies.

Category	Parameter	Value/description
Model architecture	Backbone	ResNet-50/ConvNeXt
	Initialization	ImageNet pre-trained
Optimization	Optimizer type	AdamW
	Loss function	Binary cross-entropy (BCE) loss
	Batch size	16
	Epochs	30
Learning rate scheduling	Initial learning rate	3 × 10^−5^
	Warm-up	3 epochs
	LR Scheduler	ReduceLROnPlateau (Val Loss)
	Patience	3 epochs
	Decay factor	0.1
	Minimum LR	1 × 10^−9^

### 3.3 Evaluation metrics

In light of the multi-label classification characteristics of the Topcon-MM dataset, we employ several evaluation metrics to comprehensively assess model performance: Sample Accuracy (Sample Acc), Macro F1-score (Macro F1), Macro Recall, Macro Area Under the Curve (Macro AUC), and mean Average Precision (mAP).

Sample Acc is a strict metric that requires the predicted label set to exactly match the ground truth, reflecting the model's overall consistency in multi-label prediction. To mitigate the impact of class imbalance, Macro F1, and Macro Recall are computed by averaging the performance across all categories, ensuring equal importance for each class.

In addition, Macro AUC evaluates the model's discriminative ability across different decision thresholds, while mAP measures the overall ranking performance by averaging the precision–recall characteristics over all categories. Together, these metrics provide a comprehensive evaluation of classification accuracy, robustness, and ranking capability.

The definitions of Sample Accuracy, Macro Recall, Macro F1, MacroAUC and mAP are defined in Equations 17–21, respectively:


$$\begin{array}{l} \text{Sample Acc =}\frac{\text{1}}{\text{N}}\overset{\text{N}}{\underset{\text{j = 1}}{\sum }}\left({\text{y}}_{\text{j}}={\hat{\text{y}}}_{\text{j}}\right) \end{array}$$ (17)

$$\begin{array}{l} \text{Macro Recall}=\frac{1}{C}\overset{C}{\underset{i=1}{\sum }}R_{i} \end{array}$$ (18)

$$\begin{array}{l} \text{Macro F1}=\frac{1}{C}\overset{C}{\underset{i=1}{\sum }}\text{F}1_{i}=\frac{1}{C}\overset{C}{\underset{i=1}{\sum }}\frac{2\times P_{i}\times R_{i}}{P_{i}+R_{i}} \end{array}$$ (19)

$$\begin{array}{l} \text{Macro AUC}=\frac{1}{C}\overset{C}{\underset{i=1}{\sum }}\text{AU}{\text{C}}_{i} \end{array}$$ (20)

$$\begin{array}{l} \text{mAP}=\frac{1}{C}\overset{C}{\underset{i=1}{\sum }}AP_{i} \end{array}$$ (21)

Where N denotes the number of samples, *y*_*j*_ and ${\hat{y}}_{j}$ represent the ground truth and predicted label vectors for the *j*-th sample, respectively, 𝕀(•) is the indicator function, which equals 1 if and only if the predicted labels are perfectly consistent with the ground truth, and 0 otherwise, *C* denotes the total number of categories, and *P*_*i*_ and *R*_*i*_ represent the precision and recall for the *i*-th category.

### 3.4 Comparative experiments

To verify the effectiveness and superiority of the proposed method, experiments are conducted at both single-modality and multimodal levels. The former aims to identify optimal backbone networks for each modality, while the latter validates the performance gains achieved by multimodal fusion. To ensure fairness and comparability, all methods utilize consistent dataset splits, preprocessing pipelines, data augmentation strategies, and hyperparameter settings. The experimental results are summarized in Tables 3, 4.

**Table 3:** Comparison of single-modality performance on the Topcon-MM dataset.

Modality	Methods	Macro F1 (%)	Macro recall (%)	Macro AUC (%)	Sample Acc (%)	mAP (%)
CFP	ResNet-50	73.96 ± 1.37	67.75 ± 1.46	93.88 ± 0.81	59.06 ± 1.70	83.09 ± 1.41
	Swin-T	72.31 ± 1.40	66.04 ± 1.17	93.26 ± 0.47	56.87 ± 1.29	82.58 ± 0.96
	CNX-T	72.97 ± 1.33	66.06 ± 1.41	93.34 ± 0.91	58.80 ± 1.47	83.21 ± 0.92
OCT	ResNet-50	63.43 ± 0.91	59.75 ± 1.23	90.86 ± 0.97	44.44 ± 2.12	73.79 ± 2.46
	Swin-T	65.71 ± 1.02	63.34 ± 1.40	92.13 ± 0.46	50.28 ± 1.50	75.79 ± 1.20
	CNX-T	70.23 ± 1.68	67.59 ± 0.98	93.92 ± 0.25	55.71 ± 1.49	80.76 ± 1.17

Bold values indicate the best performance among all compared methods.

**Table 4:** Performance comparison with state-of-the-art methods on the Topcon-MM dataset.

Methods	Modality	Macro F1 (%)	Macro recall (%)	Macro AUC (%)	Sample Acc (%)	mAP (%)
Concat	CFP-OCT	76.49 ± 1.32	72.61 ± 1.09	95.58 ± 0.35	61.67 ± 1.61	86.24 ± 0.99
MM-CNN	CFP-OCT	70.15 ± 1.79	63.22 ± 1.12	95.19 ± 0.24	54.55 ± 0.77	87.04 ± 0.39
CRD-Net	CFP-OCT	77.31 ± 1.84	71.72 ± 1.60	94.71 ± 0.65	59.59 ± 2.55	86.21 ± 1.67
RETFound	CFP-OCT	80.13 ± 1.01	75.38 ± 1.45	95.42 ± 0.16	60.81 ± 1.60	88.69 ± 0.29
VisionFM	CFP-OCT	81.26 ± 1.30	75.81 ± 1.31	96.00 ± 0.34	64.80 ± 1.08	91.66 ± 0.71
**Ours**	CFP-OCT	80.91 ± 0.71	76.80 ± 0.75	95.16 ± 0.53	67.32 ± 1.04	88.26 ± 0.51

Bold values indicate the best performance among all compared methods.

In the single-modality experiments, we compare several mainstream backbone networks on both CFP and OCT data, including ResNet-50, Swin Transformer Tiny (Swin-T) (27), and ConvNeXt Tiny (CNX-T) (25). As shown in Table 3, significant differences exist in the optimal model selection for different modalities. For the CFP modality, ResNet-50 achieves the best performance with a Macro F1 of 73.34%, outperforming the Transformer-based models. This suggests that convolutional structures still maintain an advantage in modeling fundus surface morphology and lesion distribution. Conversely, for the OCT modality, ConvNeXt-T performs best, reaching a Macro F1 of 70.74%. This indicates its superiority in capturing retinal layered structures and fine-grained local variations. Based on these observations, ResNet-50 and ConvNeXt-T are selected as the backbones for CFP and OCT branches, respectively.

In the multi-modal experiments, we compare our proposed method against several representative approaches, including the concatenation baseline (Concat), MM-CNN (28), and CRD-Net (20). As illustrated in Table 4, the proposed method achieves state-of-the-art (SOTA) performance across multiple key metrics.

Specifically, our method achieves a Macro F1 of 80.91%, a Macro Recall of 76.80%, and a Sample Accuracy of 67.32%, corresponding to improvements of 4.42%, 4.19%, and approximately 6 percentage points over the Concat baseline, respectively. In particular, the improvement in Sample Accuracy is consistent across all comparison settings, indicating that the proposed fusion strategy effectively enhances overall diagnostic correctness. While the Macro AUC (95.16%) shows a slight decrease compared with Concat (95.58%), this difference is marginal and primarily reflects the reduced sensitivity of ranking-based metrics in highly imbalanced multi-label settings, where classification-level improvements are more clinically relevant.

To further validate the robustness of these improvements, we conduct paired t-tests over five independent runs, as illustrated in Table 5. The results demonstrate that the proposed method achieves statistically significant improvements over MM-CNN and CRD-Net (*p* < 0.05 or *p* < 0.01) on most primary metrics, particularly Macro F1, Macro Recall, and Sample Accuracy, confirming that the observed gains are stable and not due to random variation.

**Table 5:** Statistical significance analysis using paired *t*-tests comparing the proposed method with different competing approaches.

Metric	t-statistic	*p*-value	Significance
Comparison 1: Ours vs.MM-CNN			
Macro F1	17.4314	0.000064	^**^
Macro recall	32.7824	0.000005	^**^
Macro AUC	-0.1051	0.921371	n.s.
Sample accuracy	18.7069	0.000048	^**^
Macro mAP	7.0274	0.002160	^**^
Comparison 2: Ours vs. CRD-Net			
Macro F1	4.2268	0.013404	^*^
Macro recall	7.7818	0.001470	^**^
Macro AUC	1.2362	0.283998	n.s.
Sample accuracy	6.8141	0.002424	^**^
Macro mAP	2.3084	0.082178	n.s.
Comparison 3: Ours vs. RETFound			
Macro F1	1.5987	0.185123	n.s.
Macro recall	2.0120	0.114541	n.s.
Macro AUC	–1.0649	0.346951	n.s.
Sample accuracy	6.7567	0.002502	^**^
Macro mAP	–1.3140	0.259137	n.s.
Comparison 4: Ours vs. VisionFM			
Macro F1	–0.4818	0.655152	n.s.
Macro recall	1.4247	0.227362	n.s.
Macro AUC	–2.8607	0.045896	^*^ (↓)
Sample accuracy	3.5879	0.023005	^*^
Macro mAP	–7.3700	0.001806	^**^ (↓)

Results are aggregated from five independent runs. ^*^p < 0.05, ^**^p < 0.01, n.s., not significant. (↓) denotes significant decrease.

We further extend the evaluation to stronger transfer learning baselines, including RETFound (29) and VisionFM (30). Compared with RETFound, the proposed method achieves consistent improvements in Sample Accuracy with statistical significance (*p* < 0.01), while Macro F1 and Macro Recall remain comparable, indicating similar representation capability under certain settings. For VisionFM, although it benefits from large-scale pre-training and demonstrates strong generalizable feature representations, the proposed method achieves substantially higher Sample Accuracy and comparable Macro F1 and Macro Recall. This suggests that foundation-model pre-training enhances general representation ability but does not fully align with task-specific decision optimization in the target multimodal diagnostic setting.

The relatively lower Macro AUC and mAP of our method compared to VisionFM can be attributed to the pre-training bias of foundation models, which are optimized for learning globally transferable representations rather than task-specific discriminative decision boundaries.

To further evaluate the efficiency of different methods, we report the computational complexity, memory consumption, and inference latency on the Topcon-MM dataset in Table 6. The proposed model achieves a favorable trade-off between performance and efficiency. Compared with RETFound, which contains 624.42M parameters and requires 119.38G FLOPs, our method significantly reduces computational cost while maintaining strong diagnostic performance. Even compared with VisionFM, our method achieves substantially lower FLOPs (16.94G vs. 33.73G) and faster inference speed (9.62 ms vs. 10.21 ms), while maintaining comparable memory usage. These results demonstrate that the proposed method not only improves diagnostic accuracy but also achieves superior computational efficiency, making it more suitable for real-world clinical deployment.

**Table 6:** Comparison of computational complexity and inference efficiency of different models on the Topcon-MM dataset.

Methods	Params (M)	FLOPs (G)	Memory (GB)	Time (ms)
MM-CNN	23.42	3.65	0.108	4.24
CRD-Net	128.00	10.58	0.501	13.52
RETFound	624.42	119.38	2.345	30.08
VisionFM	182.01	33.73	0.694	10.21
Ours	148.95	16.94	0.718	9.62

These results indicate that the proposed method is not only effective but also computationally efficient, making it more suitable for real-world clinical deployment scenarios where both accuracy and efficiency are critical.

### 3.5 Ablation study

To further validate the contribution of each module to the overall performance, we conduct systematic ablation experiments on the proposed MHF-Net. The architecture primarily consists of the Dual-Stream Heterogeneous Encoder (DHE), the Multi-scale Feature Enhancement (MFE) module, and the Hierarchical Contextual Fusion (HCF) module. The experimental results are summarized in Table 7.

**Table 7:** Ablation study results on the Topcon-MM dataset.

Components			Macro F1 (%)	Macro Recall (%)	Macro AUC (%)	Sample Acc (%)	mAP (%)
DHE	MFE	HCF					
✓			76.49 ± 1.32	72.61 ± 1.09	95.58 ± 0.35	61.67 ± 1.61	86.24 ± 0.99
✓	✓		79.36 ± 1.40	75.04 ± 1.93	96.09 ± 0.31	64.92 ± 1.19	88.70 ± 0.48
✓		✓	79.24 ± 1.84	75.06 ± 1.27	95.16 ± 0.83	64.86 ± 1.11	88.16 ± 0.85
✓	✓	✓	80.91 ± 0.71	76.80 ± 0.75	95.16 ± 0.53	67.32 ± 1.04	88.26 ± 0.51

Bold values indicate the best performance among all compared methods. “✓” indicates that the corresponding module is used.

The first row serves as the baseline model, which employs the DHE without MFE and HCF. As shown in Table 7, introducing the MFE module (Row 2) leads to consistent improvements in most metrics, indicating its effectiveness in enhancing feature representation.

To specifically analyze the contribution of the proposed HCF module, we perform two controlled comparisons. First, under the setting without MFE, adding HCF (Row 3 vs. Row 1) improves Macro F1 from 76.39% to 79.24% and Macro Recall from 72.61% to 75.06%, demonstrating that HCF can effectively enhance cross-modal feature interaction even without multi-scale enhancement. Second, when MFE is already incorporated, introducing HCF (Row 4 vs. Row 2) further improves Macro F1 from 79.36% to 80.91% and Sample Accuracy from 64.92% to 67.32%, indicating that HCF consistently provides complementary gains and remains effective under stronger feature representations. These results demonstrate that the proposed HCF module consistently improves discriminative performance across different settings, showing strong compatibility and robustness when combined with other components. Notably, the most complete configuration (DHE + MFE + HCF) achieves the best overall performance, with Macro F1 reaching 80.91% and Sample Accuracy reaching 67.32%, confirming the synergistic effect among the proposed modules.

To further investigate the internal design of the MFE module, we conduct additional ablation experiments by individually removing the Spatial-Guided Gating (SGG) and Cross-Scale Context Interaction (CSCI) components, as shown in Table 8.

**Table 8:** Ablation study of SGG and CSCI on the Topcon-MM dataset.

Methods	Macro F1 (%)	Macro Recall (%)	Macro AUC (%)	Sample Acc (%)	mAP (%)
w/o SGG	79.70 ± 0.47	75.69 ± 0.85	94.54 ± 0.31	65.99 ± 1.17	87.78 ± 0.76
w/o CSCI	80.23 ± 0.86	76.00 ± 1.05	94.97 ± 0.64	65.94 ± 0.53	88.01 ± 1.04
Ours	80.91 ± 0.71	76.80 ± 0.75	95.16 ± 0.53	67.32 ± 1.04	88.26 ± 0.51

Bold values indicate the best performance among all compared methods.

Removing either component leads to performance degradation across all evaluation metrics, demonstrating that both modules contribute positively to feature enhancement. Specifically, removing SGG decreases Macro F1 from 80.91% to 79.70% and Sample Accuracy from 67.32% to 65.99%. This result indicates that SGG effectively suppresses irrelevant background information and improves the discriminability of lesion-related features. Similarly, removing CSCI reduces Macro F1 to 80.23% and Sample Accuracy to 65.94%, suggesting that cross-scale contextual interaction facilitates information exchange between different feature hierarchies and improves feature consistency across scales. When both components are retained, the complete MFE module achieves the best overall performance on all metrics. These results demonstrate that SGG and CSCI play complementary roles, jointly enhancing the quality of multi-scale feature representations and contributing to improved diagnostic performance.

To further evaluate the effectiveness of the proposed fusion strategy, comparative ablation experiments are conducted with DHE and MFE fixed while replacing HCF with alternative fusion mechanisms, including concatenation, cross-attention, and channel interaction. The results are presented in Table 9.

**Table 9:** Comparison of different fusion methods on the Topcon-MM dataset.

Methods	Macro F1 (%)	Macro recall (%)	Macro AUC (%)	Sample Acc (%)	mAP (%)
Concat	79.36 ± 1.40	75.04 ± 1.93	96.09 ± 0.31	64.92 ± 1.19	88.70 ± 0.48
Cross attention	79.58 ± 0.60	74.28 ± 1.07	95.22 ± 0.35	65.47 ± 1.26	89.14 ± 0.57
Channel interaction	75.02 ± 2.21	70.23 ± 1.79	94.87 ± 0.64	59.15 ± 2.85	83.33 ± 1.76
Ours	80.91 ± 0.71	76.80 ± 0.75	95.16 ± 0.53	67.32 ± 1.04	88.26 ± 0.51

Bold values indicate the best performance among all compared methods.

Compared with simple concatenation, the proposed HCF improves Macro F1 from 79.36% to 80.91%, Macro Recall from 75.04% to 76.80%, and Sample Accuracy from 64.92% to 67.32%, indicating that progressive cross-modal interaction is more effective than direct feature aggregation. Compared with cross-attention, HCF achieves higher Macro F1, Macro Recall, and Sample Accuracy. This suggests that hierarchical interaction across both modalities and feature levels enables more effective semantic integration than single-stage cross-attention. The performance gain is even more evident when compared with channel interaction, where HCF improves Macro F1 by 5.89 percentage points and Sample Accuracy by more than 8 percentage points, demonstrating its superior ability to model complementary information across modalities. Although certain competing fusion methods achieve slightly higher values on individual ranking-based metrics such as Macro AUC or mAP, the proposed HCF consistently delivers the best overall balance across classification-oriented metrics, particularly Macro F1, Macro Recall, and Sample Accuracy, which are more directly related to diagnostic performance in multi-label disease classification.

To further assess the statistical reliability of these improvements, paired t-tests are performed based on five independent runs with different random seeds, and the results are summarized in Table 10.

**Table 10:** Statistical significance analysis using paired *t*-tests comparing the proposed HCF with different fusion strategies.

Metric	t-statistic	*p-*value	Significance
Comparison 1: Ours vs. Concat + FMS			
Macro F1	1.9430	0.1240	n.s.
Macro recall	1.8911	0.1316	n.s.
Macro AUC	–3.5945	0.0229	^*^ (↓)
Sample accuracy	5.7328	0.0046	^**^
mAP	–1.2811	0.2694	n.s.
Comparison 2: Ours vs. cross-attention + FMS			
Macro F1	5.0862	0.0071	^**^
Macro recall	5.2314	0.0064	^**^
Macro AUC	–0.2878	0.7878	n.s.
Sample accuracy	1.9096	0.1288	n.s.
mAP	–1.9166	0.1278	n.s.
Comparison 3: Ours vs. channel interaction + FMS			
Macro F1	5.0647	0.0072	^**^
Macro recall	6.1764	0.0035	^**^
Macro AUC	0.9298	0.4051	n.s.
Sample accuracy	6.4473	0.0030	^**^
mAP	7.3588	0.0018	^**^

Results are aggregated from five independent runs. ^*^ *p* < 0.05, ^**^ *p* < 0.01, n.s., not significant. (↓) denotes a significant decrease.

Compared with the concatenation baseline, HCF achieves a statistically significant improvement in Sample Accuracy (*p* = 0.0046), while maintaining competitive performance on the remaining metrics. Notably, although Macro AUC shows a statistically significant decrease, the absolute difference is relatively small, suggesting that the improvement in classification performance is not accompanied by substantial degradation in ranking ability. Compared with cross-attention, HCF yields statistically significant improvements in both Macro F1 (*p* = 0.0071) and Macro Recall (*p* = 0.0064), indicating that the proposed hierarchical fusion strategy more effectively captures complementary cross-modal information and improves class-wise recognition performance. The strongest evidence is observed when comparing HCF with channel interaction. Significant improvements are achieved in Macro F1, Macro Recall, Sample Accuracy, and mAP (all *p* < 0.01), demonstrating that the performance gains provided by HCF are substantial and consistently reproducible across repeated experiments. Overall, the statistical significance analysis confirms that the improvements achieved by HCF are not caused by random variation but result from more effective cross-modal feature interaction and semantic integration. These findings provide additional quantitative evidence supporting the effectiveness and robustness of the proposed fusion mechanism.

## 4 Discussion

**Figure 5:**
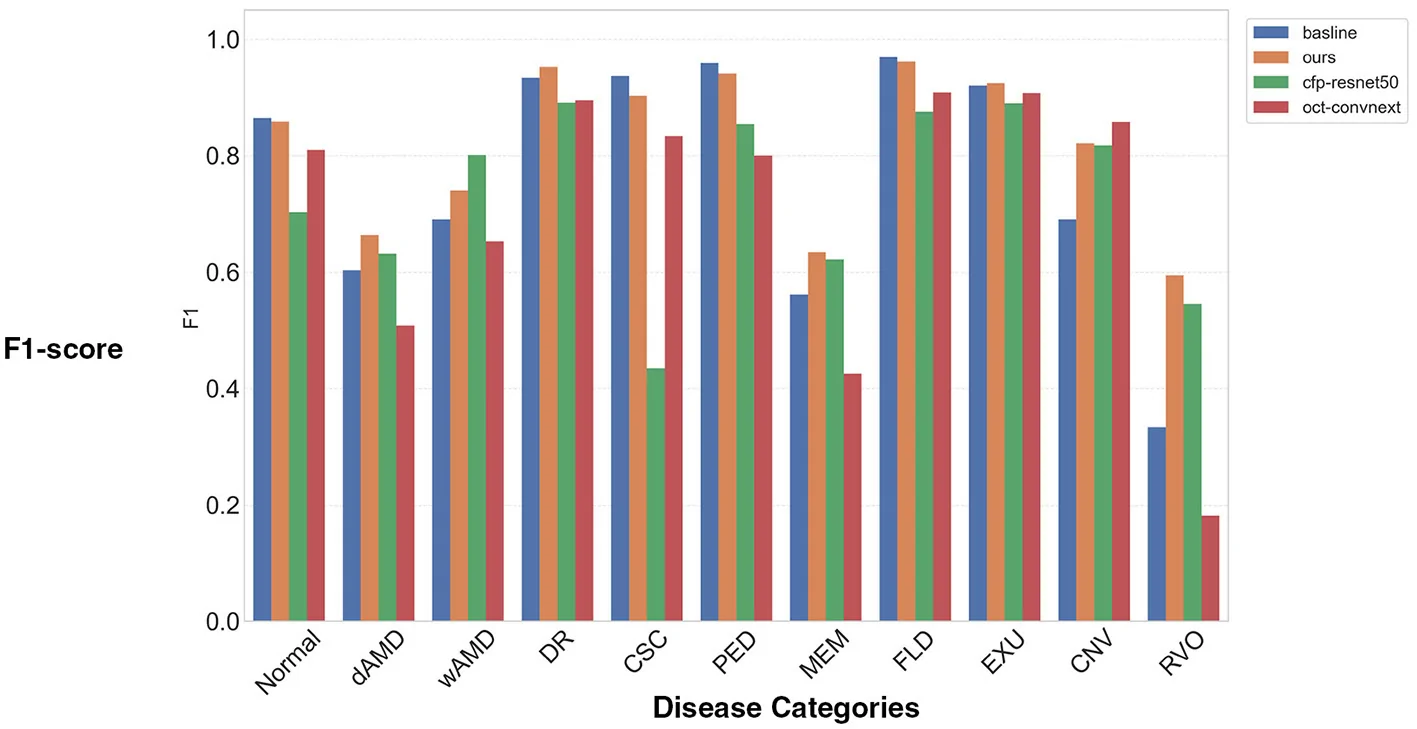
Per-category performance analysis.

To further analyze the advantages of the proposed multimodal framework, we conduct a per-category performance analysis based on the F1 scores shown in Figure 5. Overall, the proposed method achieves consistent improvements across most disease categories compared with both single-modality models and the concatenation baseline, demonstrating its effectiveness in leveraging complementary information.

Notably, for categories such as dAMD, DR, and MEM, the multimodal model outperforms all comparison methods, indicating enhanced discriminative capability through effective feature fusion. However, in a few categories (e.g., wAMD and CNV), the performance is slightly inferior to that of the dominant single modality. This suggests that, in cases with significant cross-modal discrepancies, multimodal fusion may still be affected by noise or conflicting information, highlighting the need for more robust fusion strategies.

For categories with clear single-modality dominance, such as RVO, the proposed method maintains stable performance without degradation. Unlike conventional fusion methods that may be negatively affected by less informative modalities, our approach effectively mitigates interference while preserving the contribution of the dominant modality, demonstrating improved robustness.

**Figure 6:**
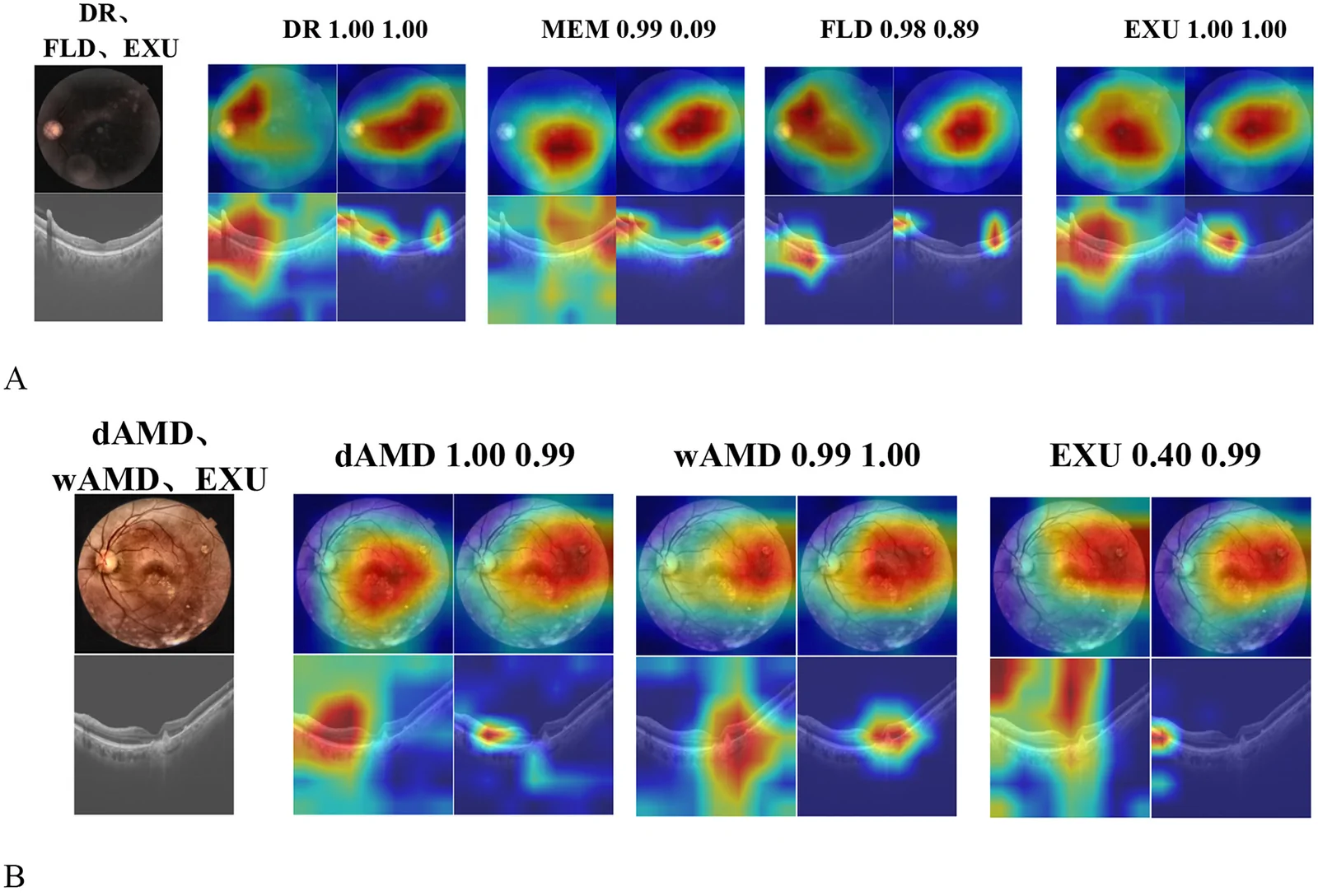
Grad-CAM++ visualization comparison between the baseline and the proposed method. **(A)** Case 1. **(B)** Case 2. For each case, the ground truth labels are shown above the original images. For each category, the heatmap on the left represents the baseline, and the one on the right represents the proposed method. The numbers above the images denote their respective prediction confidence scores.

To further validate the model's performance, Grad-CAM++ (31) visualizations are presented in Figure 6. In Case 1 (Ground Truth: DR, FLD, and EXU), the baseline model produces a false positive prediction for MEM with high confidence (0.99), accompanied by scattered and irrelevant attention regions. In contrast, the proposed method significantly reduces the confidence (0.09) and focuses on clinically relevant retinal structures.

In Case 2 (Ground Truth: dAMD, wAMD, and EXU), the baseline model fails to correctly identify EXU due to diffuse and noisy attention, whereas the proposed method exhibits more concentrated responses on lesion-related regions, leading to more reliable predictions.

In correctly classified cases, the proposed method consistently demonstrates more localized and coherent attention regions, particularly in OCT images, compared to the baseline's diffuse and noisy activation patterns. This observation further supports that the proposed framework enhances noise suppression and spatial localization, contributing to improved diagnostic reliability.

It is worth noting that the proposed Topcon-MM dataset contains 369 eyes from 203 patients, which is relatively limited for an 11-class multi-label classification task. This raises potential concerns regarding overfitting and generalization ability. To mitigate these issues, several strategies are adopted in this study. First, a patient-level splitting strategy is used to ensure that images from the same patient do not appear in both training and testing sets, thereby preventing data leakage. Second, extensive data augmentation is applied during training to increase data diversity and improve model robustness. Third, ImageNet-pre-trained backbones are utilized to provide strong feature initialization, which helps alleviate the challenges associated with limited training data.

Despite these efforts, the relatively small dataset size and single-source data collection may still limit the generalization ability of the proposed method. In particular, its performance on data acquired from different imaging devices, clinical centers, or broader patient populations remains to be further validated. Future studies should therefore evaluate the proposed framework on larger and more diverse multi-center datasets to further assess its robustness and clinical applicability.

To improve the reliability of the experimental evaluation, all experiments are repeated using multiple random seeds, and the results are reported as mean ± standard deviation. Furthermore, statistical significance analysis is conducted between the proposed method and the strongest competing baselines, providing additional evidence for the stability and robustness of the observed performance improvements.

Another limitation of the current study relates to the learning objective used for multi-label classification. We adopt the standard Binary Cross-Entropy (BCE) loss because of its simplicity and effectiveness in optimizing independent label predictions. However, BCE does not explicitly account for class imbalance or model dependencies among disease labels, which may restrict its ability to fully capture complex disease co-occurrence patterns commonly observed in ophthalmic diagnosis.

Although label imbalance is partially alleviated through patient-level data splitting and extensive data augmentation, some minority categories remain underrepresented. In addition, the proposed multimodal fusion framework improves feature discriminability by integrating complementary CFP and OCT information, which may help reduce imbalance-induced bias at the representation level. Nevertheless, these strategies do not explicitly address imbalance during optimization.

More advanced learning objectives, such as focal loss, class-balanced reweighting methods, or graph-based label dependency modeling, may further enhance performance by explicitly considering class distribution and inter-label relationships. Since these techniques are largely orthogonal to the proposed fusion architecture, investigating their integration with MHF-Net represents an important direction for future work.

## 5 Conclusion

In this paper, we proposed a Multi-modal Hybrid Fusion Network (MHF-Net) for multi-modal fundus disease diagnosis, aiming to address key challenges such as noise interference and cross-modal semantic discrepancies between CFP and OCT. The proposed framework integrated modality-aware feature learning, feature enhancement, and hierarchical fusion to effectively exploit complementary information across modalities.

Extensive experimental validation on the Topcon-MM dataset demonstrated that MHF-Net outperformed state-of-the-art single-modal and multi-modal methods across multiple evaluation metrics, confirming its effectiveness in enhancing the accuracy and stability of fundus disease diagnosis. Further ablation studies and visualization results indicated that each key module exhibited strong synergistic effects during feature enhancement and cross-modal fusion.

Overall, the proposed method demonstrated the potential of multi-modal fusion for ophthalmic image analysis. In future work, we will extend this framework to incorporate 3D OCT volumetric data and multi-modal clinical information, with the goal of further improving generalization ability and clinical applicability.

## Data Availability

The original contributions presented in the study are included in the article/supplementary material, further inquiries can be directed to the corresponding author.

## References

[1] Flaxman SR Bourne RRA Resnikoff S Ackland P Braithwaite T Cicinelli MV . Global causes of blindness and distance vision impairment 1990–2020: a systematic review and meta-analysis. Lancet Global Health. (2017) 5:e1221–34. doi: 10.1016/S2214-109X(17)30393-5 29032195

[2] Wan C Zhou X You Q Sun J Shen J Zhu S . Retinal image enhancement using cycle-constraint adversarial network. Front Med. (2022) 8:793726. doi: 10.3389/fmed.2021.793726 35096883 PMC8789669

[3] Wu J Fang H Zhu J Zhang Y Li X Liu Y . Multi-rater Prism: learning self-calibrated medical image segmentation from multiple raters. Sci Bull. (2024) 69:2906–19. doi: 10.1016/j.scib.2024.06.037 39155196

[4] Xu Y Yang W. Editorial: Artificial intelligence applications in chronic ocular diseases. Front Cell Dev Biol. (2023) 11:1295850. doi: 10.3389/fcell.2023.1295850 38143924 PMC10740206

[5] Li F Chen H Liu Z Zhang XD Jiang MS Wu ZZ . Deep learning-based automated detection of retinal diseases using optical coherence tomography images. Biomed Opt Expr. (2019) 10:6204. doi: 10.1364/BOE.10.006204 31853395 PMC6913386

[6] Ho E Wang E Youn S Sivajohan A Lane K Chun J . Deep ensemble learning for retinal image classification. Transl Vision Sci Technol. (2022) 11:39. doi: 10.1167/tvst.11.10.39 36306121 PMC9624270

[7] Li T Bo W Hu C Kang H Liu H Wang K . Applications of deep learning in fundus images: a review. arXiv preprint arXiv:2101.09864 (2021). doi: 10.48550/arXiv.2101.09864 33524824

[8] Fujimoto JG Pitris C Boppart SA Brezinski ME. Optical coherence tomography: an emerging technology for biomedical imaging and optical biopsy. Neoplasia. (2000) 2:9–25. doi: 10.1038/sj.neo.7900071 10933065 PMC1531864

[9] Yoo TK Choi JY Seo JG Ramasubramanian B Selvaperumal S Kim DW. The possibility of the combination of OCT and fundus images for improving the diagnostic accuracy of deep learning for age-related macular degeneration: a preliminary experiment. Med Biol Eng Comput. (2019) 57:677–87. doi: 10.1007/s11517-018-1915-z 30349958

[10] Wang W Xu Z Yu W Zhao J Yang J He F . Two-stream CNN with loose pair training for multi-modal AMD categorization. arXiv preprint arXiv:1907.12023. (2019). doi: 10.48550/arXiv.1907.12023

[11] Ou Z Chai W Wang L Zhang R He J Song M . M^2^ LC-Net: a multi-modal multi-disease long-tailed classification network for real clinical scenes. China Commun. (2021) 18:210–20. doi: 10.23919/JCC.2021.09.016

[12] Woo S Park J Lee JY Kweon IS. CBAM: convolutional block attention module. In: Ferrari V Hebert M Sminchisescu C Weiss Y, editors. Computer Vision – *ECCV 2018*. Cham: Springer International Publishing (2018). p. 3–19. doi: 10.1007/978-3-030-01234-2_1

[13] Li Y Daho MEH Conze PH Hajj HA Bonnin S Ren H . Multimodal information fusion for glaucoma and DR classification. arXiv preprint arXiv:2209.00979 (2022). doi: 10.48550/arXiv.2209.00979

[14] He X Deng Y Fang L Peng Q. Multi-modal retinal image classification with modality-specific attention network. IEEE Trans Med Imag. (2021) 40:1591–602. doi: 10.1109/TMI.2021.3059956 33625978

[15] Song J Zheng Y Wang J Zakir Ullah M Jiao W. Multicolor image classification using the multimodal information bottleneck network (MMIB-Net) for detecting diabetic retinopathy. Opt Express. (2021) 29:22732. doi: 10.1364/OE.430508 34266030

[16] Chen M Jin K Yan Y Liu X Huang X Gao Z . Automated diagnosis of age-related macular degeneration using multi-modal vertical plane feature fusion via deep learning. Med Phys. (2022) 49:2324–33. doi: 10.1002/mp.15541 35172022

[17] Hu J Shen L Albanie S Sun G Wu E. Squeeze-and-excitation networks. arXiv preprint arXiv:1709.01507 (2019). doi: 10.48550/arXiv.1709.01507 31034408

[18] Hua CH Kim K Huynh-The T You JI Yu SY Le-Tien T. Convolutional network with twofold feature augmentation for diabetic retinopathy recognition from multi-modal images. IEEE J Biomed Health Inf. (2021) 25:2686–97. doi: 10.1109/JBHI.2020.3041848 33264095

[19] Chen Q Keenan TDL Allot A Peng Y Agrón E Domalpally A . Multimodal, multitask, multiattention (M3) deep learning detection of reticular pseudodrusen: toward automated and accessible classification of age-related macular degeneration. J Am Med Inform Assoc. (2021) 28:1135–48. doi: 10.1093/jamia/ocaa302 33792724 PMC8200273

[20] Liu Z Hu Y Qiu Z Niu Y Zhou D Li X . Cross-modal attention network for retinal disease classification based on multi-modal images. Biomed Opt Expr. (2024) 15:3699. doi: 10.1364/BOE.516764 38867787 PMC11166426

[21] Zhou Y Yang G Zhou Y Ding D Zhao J. Representation, alignment, fusion: a generic transformer-based framework for multi-modal glaucoma recognition. In: International conference on medical image computing and computer-assisted intervention. Springer (2023). p. 704–13. doi: 10.1007/978-3-031-43990-2_66

[22] Radhi EA Mohammed MA Eid F de la Torre Díez I. Efficient blood cell classification using a hybrid variational quantum circuit-EfficientNet model on BloodMNIST. J Artif Intell Med Applic. (2025) 1:734. doi: 10.65511/jaima.v1i1.734

[23] Abdulsahib AA Mahmoud MA Aris H Gunasekaran SS Mohammed MA. An automated image segmentation and useful feature extraction algorithm for retinal blood vessels in fundus images. Electronics. (2022) 11:1295. doi: 10.3390/electronics11091295

[24] Mukhlif AA Geman O Al-Boridi O. Automated multi-class diabetic retinopathy classification using EfficientNet-B2 based on color fundus images. J Artif Intell Med Applic. (2025) 1:733. doi: 10.65511/jaima.v1i1.733

[25] Liu Z Mao H Wu CY Feichtenhofer C Darrell T Xie S. A ConvNet for the 2020s. arXiv preprint arXiv:2201.03545 (2022). doi: 10.48550/arXiv.2201.03545

[26] Wang L Dai W Jin M Ou C Li X. Fundus-enhanced disease-aware distillation model for retinal disease classification from OCT images. arXiv preprint arXiv:2308.00291 (2023). doi: 10.48550/arXiv.2308.00291

[27] Liu Z Lin Y Cao Y Hu H Wei Y Zhang Z . Swin transformer: hierarchical vision transformer using shifted windows. arXiv preprint arXiv:2103.14030 (2021). doi: 10.48550/arXiv.2103.14030

[28] Wang W Li X Xu Z Yu W Zhao J Ding D . Learning two-stream CNN for multi-modal age-related macular degeneration categorization. arXiv preprint arXiv:2012.01879 (2022). doi: 10.1109/JBHI.2022.3171523 35503853

[29] Zhou Y Chia MA Wagner SK Ayhan MS Williamson DJ Struyven RR . A foundation model for generalizable disease detection from retinal images. Nature. (2023) 622:156–63. doi: 10.1038/s41586-023-06555-x 37704728 PMC10550819

[30] Qiu J Wu J Wei H Shi P Zhang M Sun Y . Visionfm: a multi-modal multi-task vision foundation model for generalist ophthalmic artificial intelligence. arXiv preprint arXiv:2310.04992 (2023). doi: 10.1056/AIoa2300221

[31] Chattopadhay A Sarkar A Howlader P Balasubramanian VN. Grad-CAM++: generalized gradient-based visual explanations for deep convolutional networks. In: 2018 IEEE winter conference on applications of computer vision (WACV). Lake Tahoe, NV: IEEE (2018). p. 839–47. doi: 10.1109/WACV.2018.00097

